# Cellular senescence in colorectal cancer: its occurrence, effect and therapy

**DOI:** 10.3389/fonc.2025.1580951

**Published:** 2025-08-15

**Authors:** Yabing Liang, Miao Wang, Xianjue Wang, Zhiqing Yang, Shucheng Wang, Fengyi Li, Liya Su, Ling Yang

**Affiliations:** ^1^ Key Laboratory of Medical Cell Biology in Inner Mongolia, Clinical Medicine Research Center, The Affiliated Hospital of Inner Mongolia Medical University, Hohhot, Inner Mongolia, China; ^2^ State Key Laboratory of Reproductive Regulation and Breeding of Grassland Livestock, Institutes of Biomedical Sciences, School of Life Sciences, Inner Mongolia University, Hohhot, Inner Mongolia, China; ^3^ First School of Clinical Medicine, Inner Mongolia Medical University, Hohhot, Inner Mongolia, China

**Keywords:** colorectal cancer, senescence, therapy-induced senescence, senescence-associated secretory phenotype, tumor microenvironment, senolytics, therapy

## Abstract

Colorectal cancer (CRC) is one of the most common malignant tumors worldwide. Although the use of small molecule drugs or targeted drugs has shown significant efficacy in the treatment of CRC, the drug resistance after treatment and the high recurrence and metastasis rate are the key obstacles affecting the success rate of treatment and survival of patients. Cellular senescence constitutes an important barrier to tumor progression. Senescent tumor cells and stromal cells are among the reasons for cancer treatment resistance. Different senescent programs can exert inhibitory or promotional effects on CRC. In serrated adenomas of colon, the senescence induced by intrinsic oncogenes serves as a threshold that precancerous lesions must traverse to develop into cancer. And the exposing of anti-cancer treatment, such as chemotherapy and radiotherapy, some cells also enter a senescent state, presenting a stable cell cycle arrest and senescence-associated secretory phenotype (SASP). SASP can activate immune surveillance but also contribute to the maintenance of cellular senescence microenvironment to help the CRC progression. Hence, in the pursuit of effective CRC treatment strategies, the issue of senescent cells is inevitable. By targeting features of senescent cells, such as upregulated anti-apoptotic signaling, altered metabolic signaling, and differential SASP secretion, depletion of senescent cells could be a promising strategy for the treatment of CRC. This review summarizes the endogenous and exogenous factors leading to cell senescence in CRC, as well as drug mechanisms, and focuses on the research progress of senescent tumors and stromal cells in CRC. Eventually, we discuss the strategies for CRC senescent cells after anti-cancer treatment to provide some theoretical basis and direction for retarding the malignant progression and recurrence of CRC.

## 1 Introduction

Colorectal cancer (CRC) represents one of the most significant threats to human health and life. According to the World Health Organization’s cancer statistics for 2022, CRC ranks third in incidence among all cancers and second in mortality rate (1). Despite the implementation of standard screening methods and advancements in treatment strategies, both the incidence and disease-specific mortality rates of CRC have continued to decline over the past two decades. However, due to its asymptomatic nature until advanced stages, early detection remains challenging, with approximately 25% of patients diagnosed at stage IV (2, 3). Furthermore, over time, 25%-50% of patients may develop metastatic CRC (mCRC), which has a significantly lower 5-year survival rate ranging from 14% to 20%, compared to localized CRC (4). The primary treatment for CRC involves surgical resection complemented by adjuvant chemotherapy and radiotherapy. However, resistance to chemotherapy after treatment is a major challenge in the clinical treatment of CRC.The majority of patients exhibit reduced sensitivity to chemotherapy drugs in the advanced stages of treatment, leading to poor prognosis.

Cellular senescence has been described as a stable cell cycle arrest that develops in response to stress or damage (5–7). In contrast to quiescent or apoptotic cells, senescent cells cease to divide; however, they retain metabolic activity and persist in secreting factors, like pro-inflammatory cytokines, chemokines, growth factors, angiogenic factors, and extracellular proteases, collectively known as the senescence-associated secretory phenotype(SASP). The SASP exhibits autocrine and paracrine activities and is implicated not only in the chronic inflammation associated with aging but also in immune regulation and the malignant progression of cancer (8).

Cellular senescence exerts a double-edged role in cancer, contingent upon the varying environmental contexts that influence tumor progression. The accumulation of senescent cells in CRC is conducive to tumor dissemination. Rsearchs have demonstrated that a significant number of senescent cells are present in CRC tissues, including metastatic sites. In CRC with peritoneal metastasis, the peritoneal niche promotes the dissemination of senescent CRC cells, with up to 40% of these cells acquiring stem-like phenotypes and resisting most chemotherapy regimens (9). In colorectal liver metastasis (CRLM), the epithelial senescent metastatic cancer cell (eSMCC) subtype is overactivated by the oncogene c-myc, exhibiting an senescent phenotype, which promotes an immunosuppressive microenvironment (10). Senescent stromal cells also be observed in CRC progression and contribute to tumor progression. Research reported senescence in stromal cells from patients with advanced CRC receiving neoadjuvant chemotherapy, including cell-cycle arrest in fibroblasts overexpressing galactosylceramide (GALC) (11). Moreover, senescence alters the immune system, particularly affecting the proportions of adaptive immune T cells. Compared to early-stage CRC patients, those with advanced CRC exhibit more pronounced immune senescence, evidenced by a reduction in both highly proliferative CD8+ naive T cells and CD8+ central memory T cells responsible for immune surveillance (12). Therefore, cellular senescence plays a crucial role in the progression of CRC, ultimately leading to the advancement of the cancer.

In recent years, there has been an increasing number of studies investigating the occurrence of CRC related to cellular senescence and its derivative therapies. Given the current challenges in improving the clinical management and outcomes of CRC, cellular senescence may play a significant role in influencing anti-cancer strategies. This review summarizes the factors contributing to cellular senescence during CRC occurance and treatment, with a particular emphasis on the existing evidence regarding the diverse roles of senescent cells in CRC, and discusses how these characteristics can be leveraged for therapeutic purposes.

## 2 The cellular senescence characteristics and pathways

### 2.1 Cellular senescence

Senescence was first described by Hayflick and Moorhead in 1961, who observed the limited number of cell divisions, termed the “Hayflick limit”, which results from telomere shortening and is referred to as “replicative senescence” (7, 13). The progressive shortening of telomeres during cell division is interpreted by the cell as a persistent DNA damage response (DDR) aimed at preventing further genomic instability and accumulation of DNA damage (14). In addition to replicative senescence, cells may also enter a state of senescence in response to various endogenous or exogenous stimuli, and this process is independent of telomere length (15). This type of senescence that is “prematurely” evoked by cellular insults such as oncogenic activation and anti-cancer therapy, leading to oncogene-induced senescence (OIS) and therapy-induced senescence (TIS), respectively. Senescent cells exhibit distinctive features and morphological changes different from normal cells. The most widely used biomarker for senescent cells and aging is senescence-associated beta-galactosidase (SA-β-gal), which is defined as beta-galactosidase activity detectable at pH 6.0 in senescent cells. The key characteristics of senescent cells also include morphological changes, such as enlargement, flattening and increased dispersion, these are related to the decline in proliferation (16). Senescent cells also present with a state of genomic instability, telomere dysfunction, epigenetic modifications, metabolic reprogramming, and dysregulation of protein homeostasis, these characteristics are described in these reviews (5, 17, 18).

### 2.2 Pathways and molecular mechanisms of senescence

**Figure 1:**
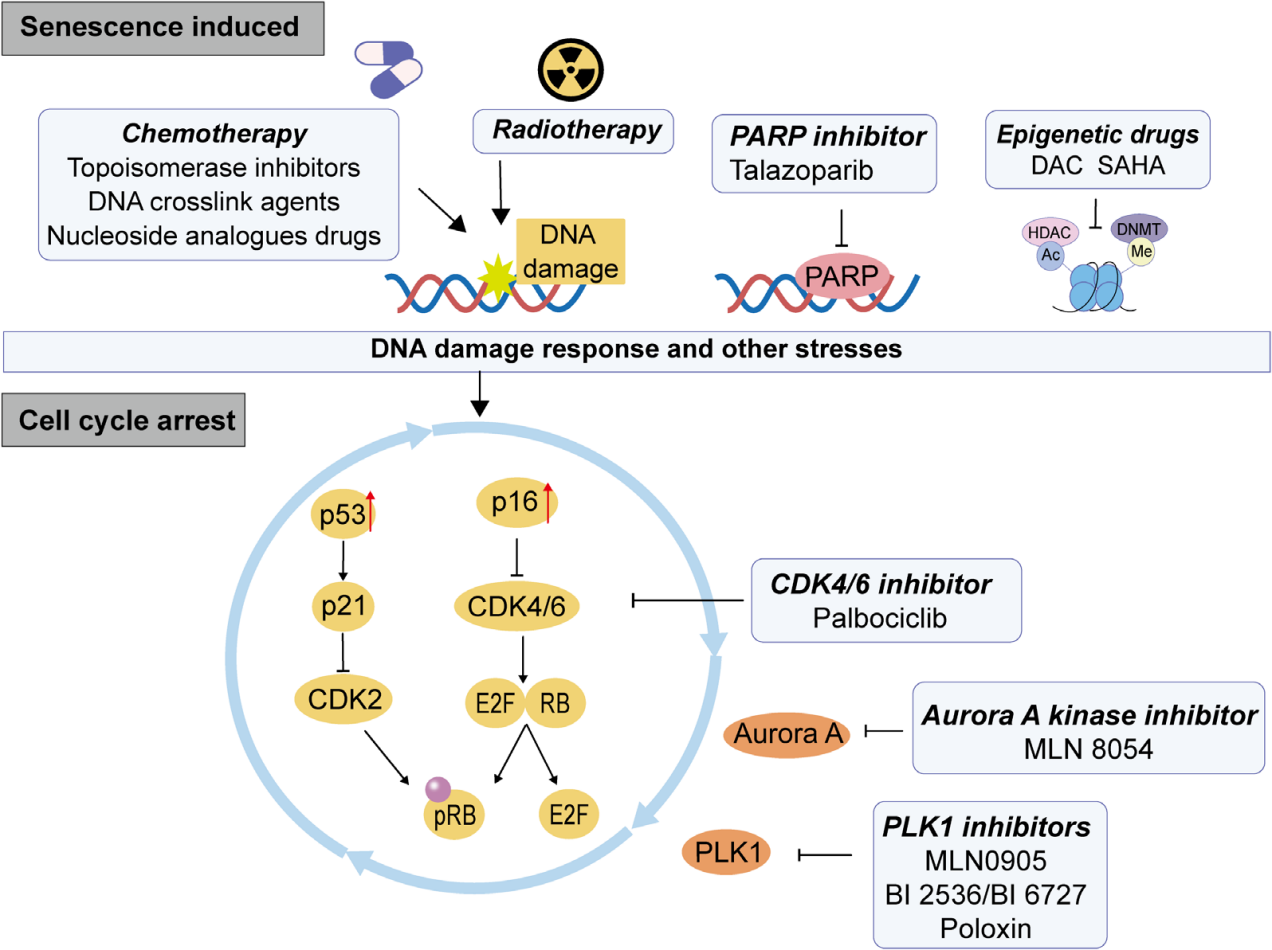
The mechanism of therapy induced senescence (TIS) in CRC. The therapeutic pressure of conventional chemotherapy, radiotherapy and targeted therapies can induce cell persistent DNA damage, which causes DDR and other stresses of DNA, then leading to the activation of p53-p21 or p16-Rb pathway, inhibiting the dephosphorylation of Rb, resulting in the inhibition of cell cycle and cell senescence.

The senescent process is mediated by signal transduction pathways, among which the p53 and Rb-controlled pathways play a crucial role. In the p53-p21 related pathway, p53 functions as a key tumor suppressor gene that is activated in response to DNA damage or other forms of cellular stress, leading to cell cycle arrest, apoptosis, or senescence. Upon activation, p53 induces the expression of the cyclin-dependent kinase (CDK) inhibitor p21. Subsequently, p21 inhibits CDK4 and CDK2 activity, thereby blocking cell cycle progression and promoting cellular senescence. In the context of the p16-Rb signaling pathway, p16 serves as a CDK inhibitor that binds to CDK4/6 and inhibits their activity, preventing phosphorylation of Rb. The non- or low-phosphorylated form of Rb can be associated with multiple transcription factors that inhibit their transcriptional activation, thus regulating cell cycle progression and differentiation. Notably, the transcription factor E2F activates critical cell cycle proteins (cyclinE and A) to initiate DNA replication. The non-phosphorylated active form of Rb binds to the transcription activation domain of the E2F, shielding its transcription activation domain and inhibiting the expression of downstream genes required for entering the S phase from the G1 phase, leading to growth arrest (19, 20) (**Figure 1**).

### 2.3 SASP

SASP is considered to be a complex secretion process composed of hundreds of different protein and non-protein signaling molecules, including a series of factors and proteins such as interleukins (IL-1α, IL-1β, IL-6, IL-8, IL-11), chemokines (CXCR2, CCL2, CXCL1, CXCL2, CXCL5, CXCL10, CXCL11), growth factors (TGFβ, GDF15, IGFBP7, IGFBP3, HGF), proteases (MMP1, MMP3), bioactive lipids, small extracellular vesicles, and non-coding nucleic acids that participate in mediating various senescence-associated biological functions in health and disease (8). SASP exhibits diversity, dynamism, and heterogeneity, influenced by the stimuli driving senescence, the specific types of senescent cells involved, and the duration of the senescent process (21, 22). Moreover, SASP significantly impacts the surrounding microenvironment, particularly in relation to tumors. For instance, SASP can either promote or inhibit tumor progression through its role in remodeling the tumor microenvironment (TME), which reciprocally influences SASP production (23). SASP also enhance immune surveillance and promote secondary cell death within cancer cell clusters (6, 24). Additionally, SASP modulates the TME by promoting angiogenesis while inhibiting the anti-tumor activity of immune cells, thereby creating an immunosuppressive TME that fosters treatment resistance and tumor advancement (25, 26). Therefore, the intricate relationship of senescent cell and TME components interplay profoundly affects tumor development.

## 3 The occurrence of cellular senescence in CRC

### 3.1 Oncogene induced cellular senescence in CRC

Oncogene-induced senescence (OIS) represents a potent intrinsic tumor suppression mechanism that can be initiated by the activation of oncogenes, such as KRAS and BRAF, alongside the inactivation of tumor suppressor genes, including PTEN and NF1. OIS suppresses the proliferation of precancerous cells and the development of tumors (27, 28). Furthermore, OIS is frequently associated with the activation of signaling pathways such as BRAF/MEK/ERK and PI3K/AKT/mTOR (20). The initial observation of OIS occurred in normal human fibroblasts expressing oncogenic KRAS; with the accumulation of p53 and p16, these cells were driven into a state of permanent G1 phase arrest (29). Research conducted in the colorectum has demonstrated that serrated lesions progressing to OIS are frequently associated with the activation of oncogenes resulting from mutations in KRAS or BRAF. Within the progress leading from precursor lesions of serrated adenomas to CRC, there is an upregulation of p16 induced senescence barriers; conversely, p16 expression is diminished during malignant transformation, suggesting that OIS serves as a protective mechanism against the progression of precursor lesions to carcinoma (30, 31).

### 3.2 Therapy induced cellular senescence in CRC

Following cancer treatment, the reduction in drug sensitivity and the emergence of resistance are significant contributors to clinical treatment failure. Recent studies indicate that exposure to radiation and chemotherapy can induce cellular senescence in both tumor cells and TME. TIS represents a form of drug resistance characterized by cell cycle arrest and the development of an SASP, which has been observed in clinical settings (32). Chemotherapy, as a systemic therapeutic approach, may result in uneven distribution of drug concentrations due to factors such as vascular architecture, immune barriers, and variations in tumor location, size, and characteristics. Although radiotherapy is localized, different regions of tumors may experience varying degrees of shielding from radiation exposure, leading to inconsistent physiological irradiation intensities. Research has demonstrated that cellular toxicity resulting from sub-toxic exposure to drugs seldom leads to apoptosis; rather, such exposures trigger genetic damage responses or oxidative stress pathways that promote anti-proliferative effects and senescent phenotype (33–35). Targeted therapy also can trigger cell senescence without causing genetic toxicity. Mechanistically, chemo or radiotherapy primarily induce cellular senescence by activating the p53/p21or p16 pathway triggered by DNA damage induced DDR (36). But different agents with distinct mechanisms of action to induce TIS (**Figure 1**), and the details are discussed below.

#### 3.2.1 Topoisomerase inhibitors

Topoisomerase (Topo) refers to a class of enzymes that catalyze the interconversion of topological isomers of DNA, thereby regulating DNA transcription, replication, and gene expression (37). Based on their catalytic mechanisms, Topo enzymes are classified into Topo I and Topo II, both of which have emerged as clinical targets for cancer therapy due to their elevated expression in tumor cells. Topo I functions by reversibly cleaving chromosomal DNA to mitigate the accumulation of abnormalities during transcription, replication, chromatin assembly, and chromosome condensation (38). Irinotecan (IRINO) is a Topo I inhibitor primarily utilized in the treatment of mCRC and other malignancies (39). Research indicates that low concentrations of irinotecan (2 µM) can induce senescence in CRC cell lines (40). Another anti-cancer agent, camptothecin (CPT), when applied to the human CRC cell line HCT116, generates Top I-mediated DNA damage, subsequently leading to cell cycle arrest and senescence (41). Topo II inhibitors exert their effects by disrupting the regulation of topological constraints within DNA and preventing the re-ligation of cleaved DNA strands, resulting in cytotoxicity and unnecessary DNA double-strand breaks that activate DDR signaling pathways (42). Doxorubicin (DOX) and etoposide are among the most commonly used topoisomerase II inhibitors for treating various cancers. Low doses of doxorubicin or etoposide are frequently employed to establish models of DNA damage-induced cellular senescence (43–47).

#### 3.2.2 DNA crosslink agents

DNA-crosslinking agents are highly reactive molecules that can form crosslinks between DNA mono-adducts, DNA-protein crosslinks, intra-strand crosslinks, and inter-strand crosslinks (48). These agents can form multiple types of DNA lesions and cytotoxicity in cells, then disrupt DNA replication and transcription, leading to the activation of DDR signaling. Platinum-based agents, including cisplatin, carboplatin, and oxaliplatin, are one group of crosslinking agents that have been used in the clinical treatment of human tumors. They primarily exert their anti-cancer effects by entering tumor cells and forming Pt-DNA complexes, which mediate tumor cell necrosis or apoptosis (49). *In vitro* treatment with high concentrations of cisplatin (CDDP) could induce apoptosis in the peripheral cell layer of a three-dimensional CRC model (multicellular spheroids), however, a significant proportion of cells within the spheroids survive this treatment. Notably, these surviving cells exhibit a high prevalence of senescent cells that show positive for β-galactosidase (50). Oxaliplatin (OXA) is a crucial component of first-line therapy for patients with advanced or metastatic CRC. Recent studies suggest that sustained endoplasmic reticulum stress and cellular senescence are key mechanisms contributing to CRC resistance to oxaliplatin (51).

Alkylating agents primarily engage with DNA by covalently attaching their alkyl groups to the N7 of guanine and the N3 of adenine within the DNA, or by forming crosslinks between DNA and proteins. This interaction disrupts DNA repair and transcription processes, thereby triggering DDR signaling (52). Temozolomide (TMZ) is a representative alkylating agent predominantly utilized for treating glioblastoma, malignant melanoma, and other solid tumors. TMZ induces GSC and CRC cells to undergo cell cycle arrest through the activation of the ATR-CHK1 axis, and sustained p53/p21 pathway. Meanwhile, TMZ activates the NF-κB pathway to promote SASP secretion (53, 54).

#### 3.2.3 Nucleoside analogues chemotherapy drugs

Nucleoside analog drugs, which are typically purine or pyrimidine analogues, belong to a group of versatile antimetabolites. Their mechanism of action involves a phosphorylation cascade that activates the drugs, ultimately forming triphosphate forms. These triphosphate forms then interact with nucleic acids, establishing complex interactions with cellular molecular components and resulting in DNA damage (55).

Fluorouracil drugs, such as 5-FU and capecitabine, are commonly used in the treatment of CRC. One of the primary mechanisms of 5-FU is the inhibition of thymidylate synthase, which is responsible for its therapeutic effects as well as side effects like severe diarrhea and intestinal injury. Research shows that repeated exposure to 5-FU can induce cellular senescence in CRC cells, leading to the development of stem cell-like properties. This may contribute to chemotherapy resistance and the recurrence of the disease following treatment (56). Additionally, 5-FU-induced senescence in endothelial cells has been linked to intestinal mucosal damage, a significant side effect related to treatment (57, 58). Researchers have also discovered that endothelial cell senescence and dysfunction can be triggered by 5-FU or serum from patients treated with capecitabine. This process involves the activation of p38 and JNK proteins, which may play a role in the cardiovascular side effects associated with 5-FU and capecitabine treatment (59). Moreover, senescence in CRC fibroblasts caused by capecitabine treatment has been shown to promote the migration and invasion of CRC cells (11).

Trifluridine (TAS-102) is a thymidine-based nucleoside analogue used to treat refractory metastatic colorectal cancer (60, 61). It is incorporated into DNA through phosphorylation, which disrupts DNA function and results in antitumor effects (62). Nucleoside analogue chemotherapeutic agents have been shown to cause DNA damage and increase DNA replication stress (DRS) in tumor cells, which can drive cellular senescence (63). Trifluridine is capable of inducing DRS in cells, leading to the accumulation of S-phase cells and the activation of the p53-p21 pathway, which in turn promotes cellular senescence and inhibits tumor cell growth (64). While it has not been confirmed whether trifluridine induces senescence in colorectal cancer (CRC) cells during treatment, it is possible that the side effects of trifluridine are related to the senescence of CRC TME cells. This connection is suggested by findings that trifluridine can induce senescence in human umbilical vein endothelial cells (HUVECs) by inhibiting autophagy, a process associated with the activation of the mTOR signaling pathway (65).

#### 3.2.4 Epigenetic agents

Epigenetic agents have been employed in cancer therapy, including DNA methyltransferase inhibitors and histone acetyltransferase inhibitors (66). Decitabine (DAC), a cytosine analog, is recognized as the most potent known DNA methyltransferase inhibitor that activates DDR signaling by inducing extensive DNA double-strand breaks or abnormal crosslinking. *In vitro* studies have demonstrated that DAC can induce senescence in human liver cancer cells and human pancreatic cancer cells (67, 68). Research indicates that CRC cells exhibit reduced clonogenic activity following exposure to decitabine; however, there is an increase in SA-β-gal activation along with upregulation of CDKN1A (the gene encoding p21), CCND1 (the gene encoding cyclin D1), and CDKN2A (the gene encoding p16) gene expression 13 days post-treatment (69). Suberoylanilide hydroxamic acid (SAHA) is a potent and reversible inhibitor of histone deacetylases (HDAC). In CRC cells treated with SAHA, some cells continue to replicate DNA without undergoing mitosis, resulting in the formation of polyploid cells that are not associated with cell division (70). Additionally, SAHA triggers glioma stem-like cells apoptosis at high concentrations (over 5 μM). In comparison, lower doses of SAHA (1 μM and 2.5 μM) inhibit glioma stem-like cells proliferation by cell cycle arrest and induce premature senescence through p53 upregulation and p38 activation (71). The published details of chemotherapy agents resulting in TIS in CRC models are summarized in **Table 1**.

**Table 1:** Senescence-inducing agents and experiment details in chemotherapy of CRC.

Class of agents	Agents	Cells/Models	Concentration	Time of treatment	Main observed results	References
Topoisomerase inhibitors	Irinotecan (IRINO)	MIP101, RKO	2 μM	48 h	Senescence induced by CPT-11 could be promoted by secreted protein acidic and rich in cysteine (SPARC), and was associated with p16 and p53.	(40)
		HCT116, SW480	2.5 μM	24 h +72 h free^1^, six cycles	Chemotherapeutics-treated CRC cells display stem-like and senescent cell features.	(56)
	Camptothecin (CPT)	HCT116	20 nM	24–96 h	High concentration (250 nM) of CPT resulted in apoptosis, low concentration (20 nM) induced senescence, and p21 was required for senescence.	(41)
	Doxorubicin (DOX)	HCT116	50/100 nM	24 h/72 h	P53 and p21 act as positive regulators of senescence-like terminal proliferation arrest	(75)
		shB7-H3-HCT116, shB7-H3-RKO	shB7-H3-HCT116: 100 nM, shB7-H3-RKO: 50 nM	96 h	B7-H3 prevented cellular senescence and growth arrest through the AKT/TM4SF1/SIRT1 pathway	(44)
		HCT116	200 nM, 400 nM	24 h	Cdk4/6 inhibitor PD0332991used with DOX augmented the number of G1-arrested cells, increase the number of colonies in DOX-induced senescence.	(43)
DNA crosslink agents	Oxaliplatin (OXA)	HCT-15	2 μM	30 days	OXA treatment induced cell senescence by persistently activated PERK and diphthamide modification of eEF2 levels,which contributed to OXA resistance.	(51)
		HCT116, SW480	5 μM	24 h +72 h free	Chemotherapeutics-treated CRC cells display stem-like and senescent cell features.	(56)
	Cisplatin (CDDP)	HCT116 spheroids	40 μM	8 days	High dose of cisplatin does not eradicate HCT116 spheroids and the surviving cells stain positive for SA-β-gal.	(50)
	Temozolomide (TMZ)	HCT116	500 μM	48 h	TMZ induced senescence dependents the p53/p21 pathway, and small molecule inhibitor NSC666715 could reduce 10-fold of the IC50 of TMZ.	(54)
Epigenetic drugs	Decitabine (DAC)	DLD-1, HT-29	1 μM	72 h	Decitabine exhibits a longer-term cytotoxic effect compared to azacitidine and can induce senescence in CRC cells.	(69)
	Suberoylanilide hydroxamic acid (SAHA)	HCT116	0.8/2 μmol/L	5 days + 5 days free	HDAC inhibitor could induce polyploidy cells senescence.	(70)
Nucleoside analogues drugs	5-fluorouracil (5-FU)	HCT116, SW480	50 μM	24 h +72 h free	Chemotherapeutics-treated CRC cells display stem-like and senescent cell features.	(56)
		HCT116, HCT-8	20 μM	48 h	The elevated expressed indoleamine 2,3-dioxygenase 1 (IDO1) in CRC cells inhibited the IGFBP5/p53 signaling pathway-mediated senescence induced by 5-FU by releasing kynurenine.	(76)

^1^Free means cells were cultured with drug-free medium.

#### 3.2.5 Radiation therapy

Radiotherapy is a widely utilized clinical modality for cancer treatment; however, when ionizing radiation deposits energy into genetic material, it can result in extensive DNA damage, typically activating the DDR through mechanisms such as double-strand breaks, single-strand breaks, and interstrand crosslinks. Inadequate repair of this damage may lead to cell cycle arrest (72). Research has demonstrated that ionizing radiation induces oxidative DNA damage in mouse models of CRC as well as patient-derived tumor organoids and primary stromal cells, resulting in p53-mediated TIS in cancer-associated fibroblasts (CAFs) (73). Moreover, ionizing radiation can induce senescence in CRC cells while reprogramming secretion and metabolic pathways (74).

#### 3.2.6 Targeted therapy

Since the p16-Rb pathway is a significant component of the senescence machinery, cellular senescence induction by CDK4/6 inhibition depends on functional Rb. CDK4/6 inhibitors, such as palbociclib (PD0332991), abemaciclib, and ribociclib, can inhibit the activity of CDK4/6 in cells, leading to cell cycle arrest in the G1 phase and thereby preventing cell proliferation (77). However, the simple administration of CDK4/6 inhibitors does not necessarily induce senescence in all cells, additional intrinsic or extrinsic factors also modulate cell fate, including downregulation of oncogene murine double minute 2 (MDM2) and inhibition of HRAS or mTOR signaling (78, 79). Palbociclib (PD0332991) was the first CDK inhibitor to be approved for the treatment of ER+/HER2- breast cancer. Palbociclib has been demonstrated to induce quiescence or senescence alone or in combination with other drugs in a variety of cell types, including breast cancer (79, 80)., melanoma (81) hepatocellular carcinoma, gastric and esophageal cancers (82), adipose sarcoma (83), leukemia, and neuroblastoma, both *in vitro* and *in vivo (* 84). In CRC related research, the use in combination of palbociclib and poly ADP-ribose polymerase (PARP) inhibitor talazoparib enhanced talazoparib-induced TIS and SASP both *in vitro* and *in vivo*, and the SASP revealed type I interferon (IFN)-related mediators which are amplified by cGAS signaling (85).

Vascular endothelial growth factor (VEGF) is regarded as a crucial mediator of tumor angiogenesis, which can mitigate the occurrence of senescence in human endothelial cells (86). The vascular endothelial growth factor receptor (VEGFR) is predominantly expressed in vascular endothelial cells (87). During the course of colitis-associated cancer, the upregulation of VEGFR2 can assist intestinal epithelial cells in bypassing cell senescence, resulting in an increase in the proliferation, migration, and survival rate of colorectal epithelial cells and promoting the development and progression of CRC. The utilization of VEGF inhibitors bevacizumab and VEGFR2 inhibitors can reduce tumor growth and promote cell senescence, which is associated with the p16 and p21 levels, respectively (88, 89).

Polo-like Kinase 1 (PLK1) is a Ser/Thr kinase that is prevalently present in eukaryotic cells and participates in the progression of the mitosis, regulating cell division, genome stability, DDR, and so forth (90). Research has demonstrated in multiple cell types, including CRC cells, that PLK1 inhibitors might partially induce mitotic arrest by causing the accumulation of unrepaired double-strand breaks, thereby leading to cell senescence (91). In addition, PLK1 inhibitors could induce HCT116 p21+/+ cell senescence, but massive apoptosis was induced by PLK1 inhibitors in HCT116 p21-/- cells (92). Another Ser/Thr kinase, Aurora A kinase, which also plays an important role in mitosis, has been found to be overexpressed in tumors. Targeting Aurora kinase is a promising therapeutic target in cancer treatment (93). Research has shown that inhibition of Aurora A kinase can induce cell senescence in various cancers (94, 95). For example, MLN8054 induces senescence phenotypes in cultured cells and in *in vivo* models, including enlargement of cell bodies/nuclei, increased vacuolization, positive SA-β-gal, upregulation of p53 and p21, and a low phosphorylated Rb state (96). The details used in the current CRC cellular senescence-induced by targeted therpies are summarized in **Table 2**.

**Table 2:** Senescence-inducing agents and experimental details in targeted therapy of CRC.

Class of agents	Agents	Models	Concentration	Time of treatment	Main observed results	References
CDK4/6 inhibitors	Palbociclib (PD0332991)	HCT116, HT26	1 μM	48–72 h	Talazoparib and palbociclib combination induced senescence and IFN mediate SASP, and established a antitumor TME.	(85)
PARP inhibitors	Talazoparib	HCT116, HT26	0.5 μM	24–72 h		(85)
VEGF/VEGFR inhibitors	Bevacizumab	MIP101, RKO, SW620, SW480	10 μg/mL, 50 μg/mL	NA	The senescence induced by bevacizumab is associated with the upregulation of p16, but is independent of p53.	(88)
	AZD-2171	HCT116	500 nM	NA	Inhibition of VEGFR2 signaling leads to senescence of CRC cells.	(89)
PLK1 inhibitors	MLN0905	HCT116	50 nM	5–20 days	Plk1 inhibition induced senescence partially due to the presence of DNA double-strand breaks in mitosis.	(91)
	BI 2536/BI 6727	HCT116	25 nM	96 h	PLK1 inhibitors could induce p21 increased, and long-term treatment with PLK1 inhibitors induced senescence of tumor cells with functional p21.	(92)
	Poloxin	HCT116	25 μM	96 h		(92)
Aurora A kinase inhibitors	MLN8054	HCT-116, HCT-116 xenografts	0.25, 1, 4 μmol/L 30 mg/kg	5, 14 days 21 days	Inhibition of Aurora A could induce tumor cell senescence both *in vitro* and *in vivo*.	(96)

NA indicates not mentioned in the article.

## 4 The effect of senescent cells in CRC

**Figure 2:**
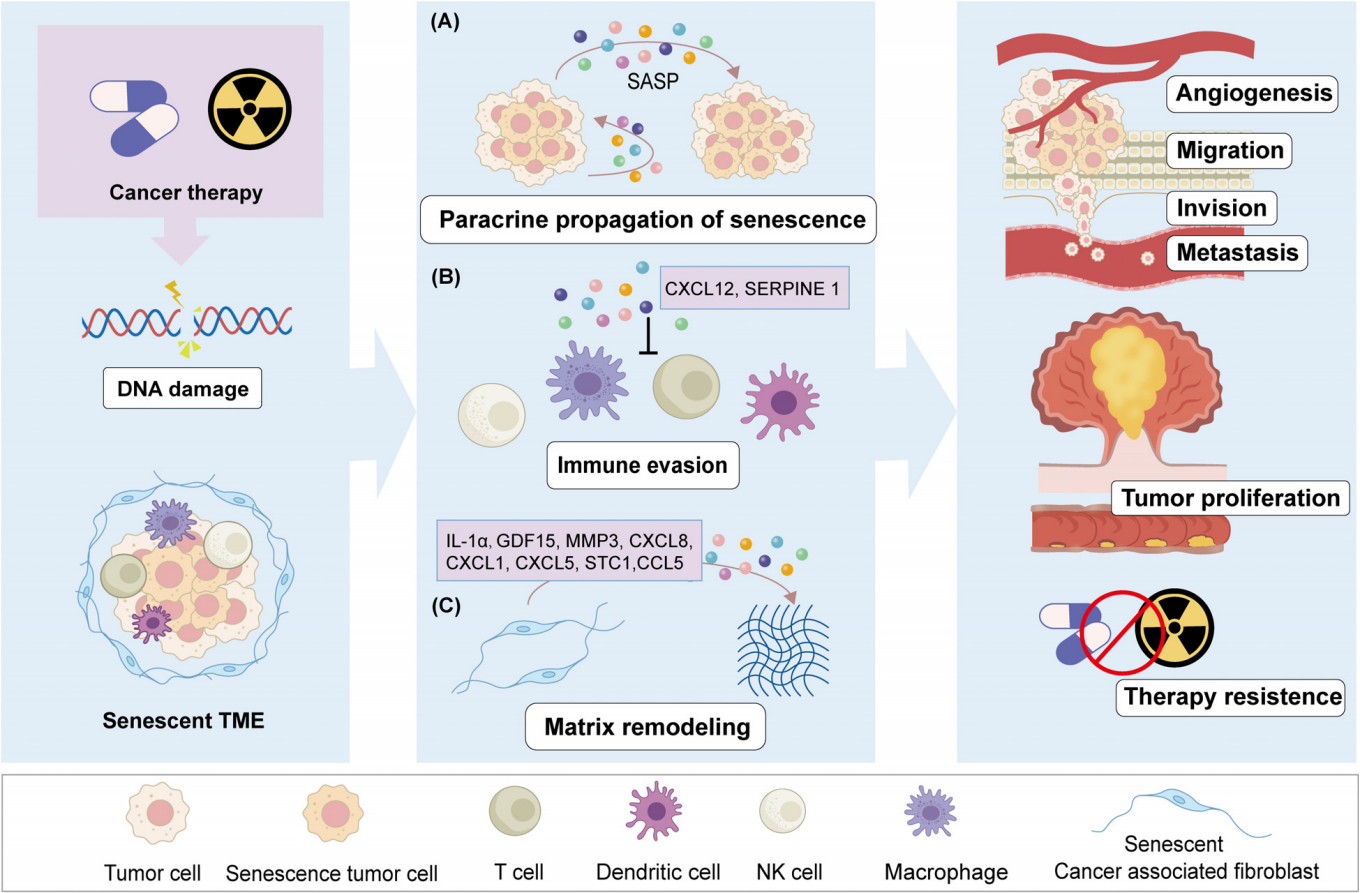
The negative effect of therapy-induced senescence in CRC. After chemo- or radio-therapy stress, CRC cells undergo DNA damage, leading to a cellular senescence phenotype and senescent TME. Senescent cells through secrete SASP mediate the tumor progression. SASP factors induce paracrine senescence of adjacent cancer cells through autocrine and paracrine signals **(A)**.SASP factors promote tumor angiogenesis and preventing the antitumor effects of immune cells **(B)**. Therapy induced senescent CAFs regulate and remodel the TME through SASP **(C)**. These effects of senescent cells promote cancer cells proliferation, migration, and invasion, lead to CRC metastasis or recurrence, as well as resistance to therapy.

Intercellular interactions in the TME primarily occur through direct contact and paracrine signaling. In the context of cellular senescence, these senescent cells release heterogeneous SASP factors that influence intercellular communication within the TME, thereby sustaining a senescent microenvironment conducive to tumor initiation and progression (**Figure 2A**).

### 4.1 Senescent tumor cells regulate CRC immune response

During the progression of cancer, different types of senescent cells will appear and coexist in the TME (97). Senescent tumor cells (STCs) can accumulate within tumors, which are the most prevalent following anti-cancer therapies. These STCs are known to secrete a diverse array of SASP associated with cancer invasion and immune evasion (98, 99). The functional state of T cells within the TME is a critical determinant of effective anti-tumor immunity and immunotherapy. Research has demonstrated that senescent CRC tumor cells inhibit the infiltration of CD8+ T cells by secreting elevated levels of chemokine ligand 12 (CXCL12), resulting in the loss of chemokine receptor (CXCR4) on T cells and impairing their directed migration (100). Furthermore, STCs facilitate the differentiation of monocytes into M2 macrophages through the secretion of colony stimulating factor 1 (CSF1), which subsequently inhibits CD8+ T cell activation. The increased expression of CXCL12 and CSF1 in senescent CRC tumor cells establishes a cytokine barrier that suppresses immune infiltration by tumor-associated T cells, thereby protecting non-senescent tumor cells from immune attack and promoting resistance to CRC (100) (**Figure 2B**). In addition, secreted Phosphoprotein 1(SPP1) is a secreted cytokine closely associated with tumorigenesis, invasion, and metastasis (101, 102).Single-cell transcriptomic analyses also revealed the presence of a high-expressing SPP1+ macrophage subpopulation in high-grade CRC, characterized by robust SASP features and serving as a critical component of the TME that contributes to tumor senescence (103).

Most studies focus on STCs and their role in promoting tumor immune evasion and progression. However, recent research suggests that chemotherapy-induced tumor senescence can actually activate anti-tumor immunity. For example, in breast cancer mouse models, CDK4/6 inhibitors like abemaciclib and palbociclib were found to induce TIS, which enhanced the tumor’s immunogenicity by improving antigen presentation and reducing the proliferation of regulatory T cells (Tregs). This, in turn, promoted the clearance of tumors by cytotoxic T cells, with similar findings noted in CRC models (104). Moreover, senescent cells induced by the combined therapy of talazoparib and palbociclib promoted the activation of CD8 T cells and natural killer (NK) cells, and the decrease of macrophages and granulocytic myeloid-derived suppressor cells (G-MDSCs) in the TME. This enhanced the efficacy of anti-PD-L1 treatment in immunocompetent mice (85). Additionally, immune responses activated by STCs have been observed in various other malignancies. In multiple myeloma mouse models, TIS increased the surface expression of NK cell receptor ligands, such as NKG2D (RAE-1, MICA, MULT-1) and DNAM-1 (PVR), enhancing NK cell recognition and tumor cytotoxicity (105). Moreover, an Aurora kinase inhibitor MLN8237 induced TIS and triggered an NF-κB-associated SASP in human melanoma xenografts. This led to the recruitment of macrophages and neutrophils, which helped mediate immune surveillance and inhibit the growth of senescent tumor cell implants (106). These findings highlight the potential of TIS to stimulate anti-cancer immunity. However, the effects of senescent cells on the immune system can be complex. Whether they exert pro- or anti-tumor effects depends on several factors, including drug dosage, the burden of senescent cells, and dynamic changes in the SASP. These variables collectively influence how immune cells are functionally polarized, affecting their activation states and subtype compositions.

### 4.2 Senescent stromal cells promote CRC progression

Senescent stromal cell is a common phenomenon in CRC and intricately linked to tumor progression. CAFs represent a key stromal cell component of the TME and play a critical role in cancer progression. The niche of senescent cells also shows enhanced epithelial-mesenchymal transition (EMT)-related signals in CRC patients with therapy (56, 107).Growth differentiation factor 15 (GDF15) could enhance EMT by activating the Smad2 and Smad3 pathways through interaction with TGF-β receptors (108). Research has demonstrated that the quantity of senescent cells in the colons of CRC patients is elevated, with senescent fibroblasts secreting GDF15 as an essential SASP factor. GDF15 contributes to a microniche conducive to CRC initiation and progression via the MAPK and PI3K signaling pathways within the colon (109). Following this, researchers identified key SASP genes—GDF15, MMP3, CXCL8, CXCL1, CXCL5, STC1, and CCL5—as “core senescent features” for identifying senescent fibroblasts in the colon (22) (**Figure 2C**). Studies have demonstrated that the overexpression of phospholipase D2 (PLD2) in CRC cells could induce senescence in adjacent fibroblasts. The senescent fibroblasts, in turn, respond to extracellular PLD2 by enhancing the secretion of SASP factors, which promotes tumor growth and the stem cell-like properties of CRC cells through activation of the Wnt signaling pathway (110). In CRC patients treated with oxaliplatin and capecitabine, senescent fibroblasts exhibiting overexpression of galactosylceramidase (GALC) were observed to promote the tumor progression by up-regulating the transcription factor ATF3 (11).

Senescent fibroblasts accumulate in tissues affected by senescence and oxidative stress and have been demonstrated to promote CRC development and drug resistance through IL-1 (73, 111). The key SASP factor, IL-1α, can stimulate the senescence-related secretion of IL-6 and IL-8 via the IL-1R signaling pathway. Both IL-6 and IL-8 are pro-inflammatory cytokines that enhance growth arrest in senescent cells and promote cancer cell invasion into the basement membrane, thereby facilitating tumor metastasis (112). Notably, Low levels of IL-1 signaling pathway receptor antagonist IL-1ra signal indicate that IL-1 signaling is activated in CRC patients, making CAF susceptible to TIS. Furthermore, inhibiting IL-1 signaling or employing preventive or eliminate senescent of polarized inflammatory CAFs (iCAFs) under therapy stress could increase sensitivity to radiotherapy (73).

### 4.3 Senescent endothelial cells promote CRC angiogenesis

The process of angiogenesis is a critical event in the advancement and metastasis of cancers. As tumors exceed a certain size, blood vessels facilitate the delivery of oxygen and nutrients to the TME, thereby supporting tumor growth, invasion, and metastasis. Zheng and colleagues analyzed published transcriptome datasets, revealing that endothelial cells display the highest levels of cellular senescence across various cancer types compared to tumor cells or other cell types within the tumor vasculature (113). Furthermore, senescent endothelial cells are positively associated with pro-tumor signaling pathways, dysregulated immune responses that enhance tumor proliferation, and increase poor prognosis in cancer patients (113). In the CRC treatment with 5-FU, intestinal damage was induced by senescence of intestinal epithelial cells and inflammation and oxidative stress in treatment (57). In another research, doxorubicin enhanced the transcription of SASP gene chemokine ligand 2 (CCL2) by activating NF-κB, thereby facilitating the growth and invasion of CRC cells both *in vitro* and *in vivo*. Conversely, exogenous administration of the classical anti-aging protein Klotho effectively suppressed the expression of SASP factors, including CCL2, thus attenuating CRC progression induced by senescent stromal cells (114).

In conclusion, although the pressure of treatment can eliminate most malignant cells, the pressure on the remaining cells in the microenvironment will also lead to cell senescence, and the senescent cells can help tumor cells escape immune surveillance and change and remodel the microenvironment, which in turn brings opportunities for CRC recurrence and metastasis, and also brings obstacles to the continuous effectiveness of treatment (**Figure 2**).

## 5 Senotherapeutics in CRC

As previously mentioned, the persistence of senescent cells after treatment is detrimental, prompting the exploration of strategies to eliminate these cells through senotherapeutics, minimizing the risk of tumor progression and bringing new treatment opportunities to cancer therapy. In preclinical models, interventions targeting persistent senescent cells post-treatment have demonstrated efficacy in delaying, preventing, or alleviating a range of diseases (115, 116). Senotherapeutics can be categorized into two classes: senolytics, which selectively kill senescent cells or induce senolysis, and senomorphics, which attenuate the pathological SASPs to cause senostasis.

### 5.1 Senolytics

It is currently recognized that senescent cells exhibit a stable phenotype characterized by distinct metabolic, secretory, transcriptional, and epigenetic profiles, which reveal heightened selectivity for exposed these vulnerabilities. The weaknesses of senescent cells—such as alterations in apoptosis regulation—can serve as selective targets for senolytic agents. Senolytics primarily facilitate the apoptosis of senescent cells by targeting key enzymes involved in pro-survival and anti-apoptotic pathways, including p53, p21, Bcl-2 family proteins, Akt, PI3K, and FOXO4 (117). Senescent cells often have elevated levels of anti-apoptotic BCL-2 family proteins, which represent a primary focus of research for drugs targeting these senescent cells in senolytic therapy. Navitoclax (also known as ABT-263) is a BH3 protein mimetic and selective inhibitor of the Bcl-2, Bcl-w, and Bcl-XL, which re-activate the apoptotic pathway to eliminate senescent cells (118, 119). Research has demonstrated that the expression level of RNA polymerase I (RNAPOL1) is significantly elevated in CRC. Targeting RNAPOL1 with the specific inhibitor CX5461 disrupts nuclear integrity, induces imbalances in ribosomal proteins, and results in irreversible growth arrest characterized by features of senescence and terminal differentiation. The effects of CX5461 or navitoclax on CRC cell viability are minimal when administered individually; however, continuous combination treatment with both agents substantially reduces CRC cell viability (120). Navitoclax has been evaluated as a senolytic agent in multiple clinical trials for the treatment of solid tumors (e.g., NCT01009073, NCT05358639, NCT00878449). In a phase I-II clinical study (NCT02079740), navitoclax was administered in combination with the MEK inhibitor trametinib for the treatment of solid tumors harboring KRAS and NRAS mutations; however, no partial responses (PR) were observed among CRC patients (121). A prevalent adverse effect observed in navitoclax clinical trials is severe thrombocytopenia (122), which has also limited its clinical application. Venetoclax (ABT199) was developed to address the specificity and the side effect of thrombocytopenia, a re-engineering of navitoclax. Venetoclax could inhibit the growth of Bcl-2-dependent tumors *in vivo* and spares human platelets (123, 124). Targeting senescent CRC cells with venetoclax can enhance the tumor’s sensitivity to radiation when combined with radiotherapy (73).

Among the senolytic agents utilized in the treatment of cancers, quercetin and fisetin are naturally flavonoid compounds, with quercetin recognized as the first identified anti-senescence drug. Research indicates that quercetin promotes apoptosis by interacting with PI3K isoform and Bcl-2 family members. Furthermore, fisetin exhibits superior senolytic activity compared to quercetin (118). Studies have shown that gamma radiation induces growth arrest in CRC cells, elevates the expression of p16 and p21, and increases the positive rate of SA-β-gal. Subsequently, treatment with fisetin, quercetin or navitoclax could effectively clear senescent cells. The combination of these agents with radiation significantly reduces the expression levels of p16 and p21 while synergistically enhancing CRC cells death compared to single use radiation (125).

Heat shock protein 90 (HSP90) is a highly expressed chaperone protein family, with many members involved in cell proliferation, angiogenesis, and tumorigenesis. Consequently, the inhibition of HSP90 represents an effective strategy for cancer treatment. Several HSP90 inhibitors, including Geldanamycin, 17-AAG (tanespimycin), and 17-DMAG (alvespimycin), have demonstrated senolytic activity. Mechanistically, HSP90 inhibitors such as 17-DMAG can promote apoptosis in senescent cells by disrupting the interaction between HSP90 and Akt, thereby inhibiting the active form of Akt (126). Coroglaucigenin (CGN), a natural product isolated from calotropis gigantean, exhibited an activity of HSP90 inhibitor, which disrupted the association of HSP90 with both CDK4 and Akt, leading to CDK4 degradation and Akt dephosphorylation, eventually resulting in senescence and autophagy in CRC cells (127). In addition to small molecule compounds, the heterogeneity of senescent cells can also serve as an immunogenic stimulus to stimulate anti-tumor immunity for clearing senescent cells. For instance, β2-microglobulin (B2M), a cell membrane marker widely expressed in aging tissues, and antibodies bound to doxorubicin could kill doxorubicin-induced senescent CRC cells (128).

### 5.2 Senomorphic

Senomorphic is recognized as an adjunct or alternative therapy for anti-aging interventions. It functions by regulating the key signal pathways of SASP from senescent cells, mitigating their pro-inflammatory characteristics, and inhibiting detrimental SASP components without inducing cell death (15). Notable examples of such agents include rapamycin, metformin, resveratrol, and aspirin. mTOR is an essential regulator of the SASP, and mTOR inhibitors have shown senomorphic effects in senescent cancer cells (129). Rapamycin, an mTOR inhibitor, disrupts the mTOR signaling pathway to attenuate NF-κB activity at the translational level and inhibit pro-inflammatory SASP factors. Treatment with rapamycin (and its analog RAD001) has been shown to diminish carcinogenic SASP and mitigate age-related conditions (130). KRAS serves as a therapeutic target in CRC, and the clinical application of KRAS inhibitors in combination with EGFR inhibitors has shown promise for CRC treatment. Studies indicate that activating the mTOR signaling pathway occurs when cells exhibiting acquired resistance to therapy are re-exposed to the same drug or when patients experience KRAS amplification following drug withdrawal. This activation results in senescence-related phenotypes within the cells, ultimately leading to secondary resistance. Notably, these resistant cells demonstrate increased sensitivity to the mTOR inhibitor AZD8055. The treatment using AZD8055 or ABT-263 effectively suppresses the proliferation of drug-resistant cells post-treatment cessation (131).

**Figure 3:**
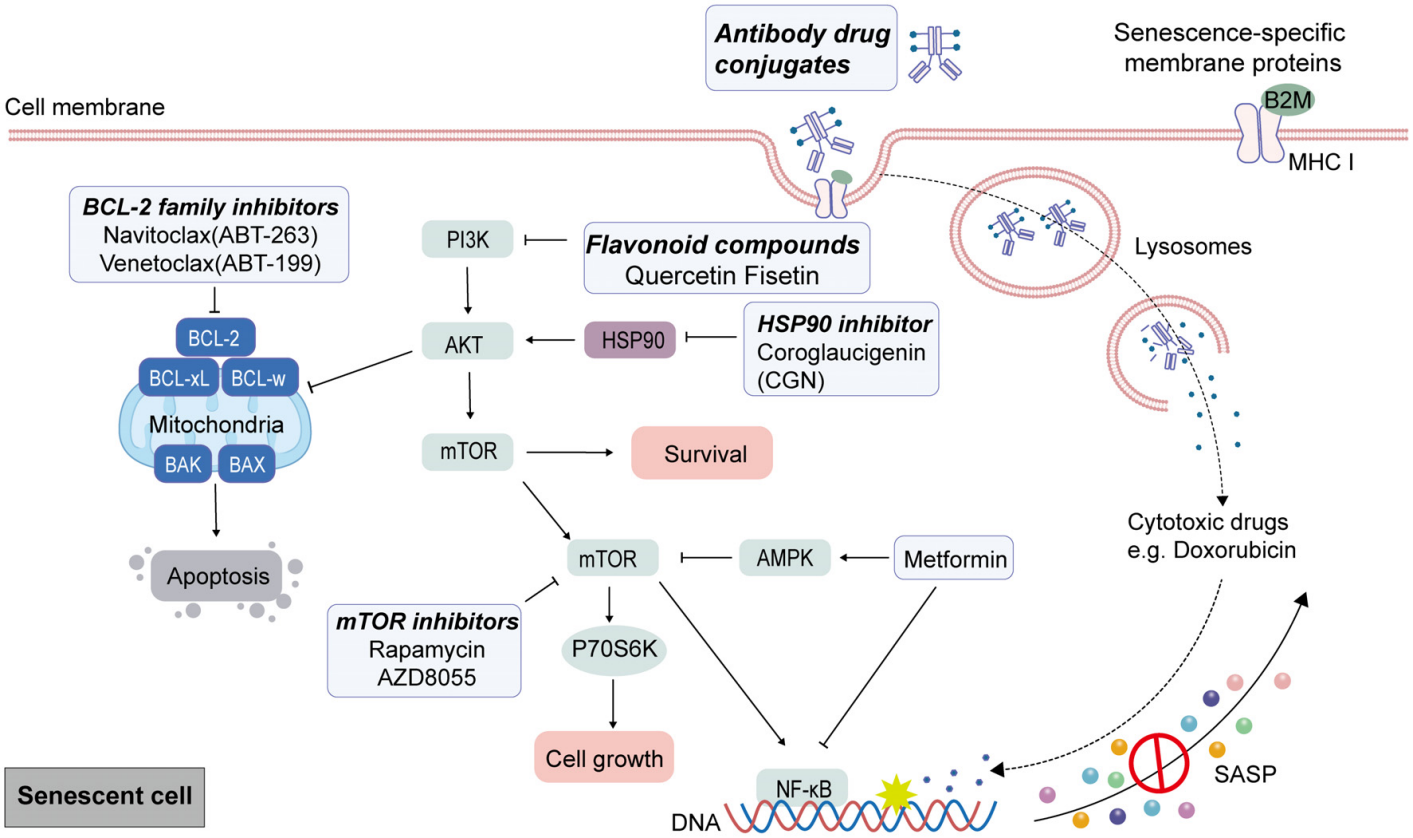
Target senescent cells by senolytic and senomorphic therapies in CRC. After senescence caused by cancer therapies as the first punch, senescent characteristic can be used to eliminate tumor cells by senolytic and senomorphic therapies. Anti-apoptotic Bcl-2 family proteins are up-regulated in senescent cells, and endogenous apoptosis can be induced by BCL-2 family inhibitors. Quercetin and Fisetin interfered with the anti-apoptotic protein BCL-xL by inhibiting upstream pathways including Pl3K. HSP90 inhibitors promote apoptosis in senescent cells by disrupting the interaction between HSP90 and AKT. Senescent cells show changes in surface membrane protein expression. Cytotoxic compounds are delivered by monoclonal antibody-drug complexes that recognize and bind to a specific antigen of the surfaceome of senescent cells to kill senescent cells. The mTOR inhibitors, Rapamycin and AZD8055, through disrupt the mTOR signaling pathway to attenuate NF-κB activity at the translational level and inhibit pro-inflammatory SASP factors. The inhibitory effect of metformin on senescent cells is partly mediated by the activation of AMPK signaling to inhibit mTOR activation and the inhibition of NF-κB.

Metformin is a lipophilic biguanide that inhibits hepatic gluconeogenesis and improves peripheral utilization of glucose. Metformin has been used in various age-related disorders, including insulin resistance, obesity, liver diseases, cardiovascular diseases, and neurodegenerative diseases (132, 133). In recent years, a number of data have shown that metformin is effective in suppressing cellular senescence and SASP in cancer treatment (134–136). A potential anti-tumorigenic effect of metformin may be mediated by its role in activating AMPK, which in turn inhibits the mTOR. Epidemiological studies have demonstrated that type 2 diabetes is associated with poor prognosis in CRC patients (137). Melia and colleagues reported that type 2 diabetes promotes colorectal tumorigenesis by inducing premature cellular senescence (111). During treatment, intestinal damage induced by 5-FU correlates with the accumulation of senescent cells (57, 138). Metformin can inhibit the mTOR/p70S6K pathway both *in vitro* and *in vivo* to mitigate oxidative stress and delay the senescent of intestinal epithelial cells, thereby significantly reducing the side effects associated with drug-induced intestinal injury (138). Additionally, metformin inhibited the expression of various inflammatory cytokines secreted during cellular senescence associated with the SASP, a process mediated by IKK/NF-κB activation, thereby suppressing the proliferation of tumor cells stimulated by conditioned media from senescent cells (139). Moreover, metabolic reprogramming significantly contributes to the premature senescence of T cells within the TME, as T lymphocytes exhibit heightened sensitivity to tryptophan deficiency, which can induce cell cycle arrest in the G1 phase (140). Metformin could modulate tryptophan metabolism by reducing its metabolic activity in CRC cells while enhancing tryptophan metabolism in CD8+ T cells. In contrast, CRC cells not treated with metformin outcompete CD8+ T cells for available tryptophan, resulting in impaired CD8+ T cell function (141). In summary, metformin treatment diminishes the uptake of tryptophan by CRC cells, thereby restoring its availability to CD8+ T cells and augmenting their cytotoxic efficacy. The mechanisms of Senolytics and Senomorphic therapies in CRC are described in **Figure 3**.

### 5.3 Senotherapy for CRC

In cancer therapy, a sequential “one-two punch” strategy is employed: first, anticancer agents induce tumor cell apoptosis (potentially triggering residual cell senescence), followed by senolytic agents to eliminate senescent cells or senomorphics to suppress SASP (142). In this way, on the one hand, it can prevent tumor progression, recurrence and drug resistance, thereby avoiding the harmful accumulation of senescent cells within the tumor; at the same time, on the other hand, it can reduce treatment side effects by removing senescent cells from normal tissues.

As mentioned earlier, some therapeutic strategies employing “one-two punch” approaches to jointly target senescent cells have been explored in preclinical CRC models, and currently several clinical trials are underway. In a phase I/II clinical trial (NCT00409994), the safety, antitumor activity, and metabolic effects of rapamycin combined with short-course radiotherapy were evaluated in rectal cancer (143). While significant metabolic responses to rapamycin were observed, the treatment cohort did not show a notable increase in pathological complete response (pCR). Additionally, several clinical trials have assessed the clinical benefits of metformin in combination with chemoradiotherapy (NCT05921942, NCT01941953, NCT01930864, NCT02473094), neoadjuvant therapy (NCT03053544, NCT02437656), or targeted therapy (NCT03800602) in advanced CRC patients, yet only a subset of patients demonstrated modest benefits (144–147). Although these trials utilized drugs with senomorphic properties, they did not evaluate the effects of senotherapies from a cellular senescence perspective. For example, they did not examine biomarkers of senescent cells or levels of the SASP in treated tissues. However, given the complexity and variability of SASP depending on treatment modalities and disease stages, there is currently no unified pan-senescence biomarker to delineate the senescent cell state. This highlights the challenges in clinically assessing patient benefits through senotherapies aimed at eliminating TIS cells.

Given these challenges, researchers are exploring various *in vivo* methods to assess cellular senescence for dynamically tracking the senescent state of individuals or cells. For instance, Jessie and colleagues developed highly tissue-penetrable probes based on senescence-associated biomarkers (e.g., β-galactosidase and lipofuscin) to selectively detect senescent cells using nanoparticles, as well as novel chemical approaches for prodrug design that can both detect and eliminate senescent cells, which have been tested and evaluated *in vitro* and *in vivo*, providing potential strategies for clinical monitoring and tracking senotherapeutic interventions. These methods have been comprehensively reviewed (148). Additionally, 3,4-Cyclohexenoesculetin beta-D-galactopyranoside (S-Gal) and its analog C3-GD can be used for MRI-based detection of β-galactosidase activity *in vivo (* 149, 150). Similar non-invasive techniques for evaluating β-galactosidase activity hold promise for clinical translation. However, although β-galactosidase is one of the primary biomarkers for assessing cellular senescence, its elevated activity is not exclusive to senescent cells (151), limiting its specificity as a senescence marker.Therefore, it is necessary to develop novel methodologies, identify and establish more specific senescence biomarkers, and explore more accurate detection and therapeutic strategies targeting cellular senescence.

## 6 Senescence escape

The traditional definition of cell senescence is the occurrence of irreversible cell cycle arrest while still maintaining active metabolism. However, this concept has been challenged as some studies have shown that some senescent cells can escape proliferation arrest and re-enter the cell cycle, a phenomenon known as “senescence escape” (152). Among them, TIS-induced cells were found to exhibit stem cell-like phenotype, which may be one of the important reasons why cells escape senescence after the drug is removed. Moreover, tumor cells that escape from senescence often have more proliferative potential and malignant phenotypes, this may be an important factor in promoting tumor recurrence and treatment related resistance. A study showed TIS HCT116 cells treated with autophagy inhibitor Bafilomycin A1 (Baf1A) to re-activate its proliferative potential (153). Prior to escaping, these escaped cells exhibit a distinct phenotype characterized by enhanced self-renewal ability achieved through upregulation of stem cell-related genes, like increased NANOG expression, CD24+ population, and colony formation (153). In contrast to the pro-oxidative micro-environment linked to senescence, these cells possess a reduced level of reactive oxygen species (ROS) and an elevated antioxidant phenotype. A study using TIS-escaped breast cancer cells demonstrated a redox environment with low ROS and increased antioxidant enzymes expression (154). This low ROS environment and high Oct-4 level and CD133 appear like cancer stem cells. Research has demonstrated that cells released from senescence can re-enter the cell cycle with a vigorous proliferation and Wnt-dependent clonal growth potential and display a higher tumor initiation potential. These temporarily senescent cells reprogram non-stem cells into self-renewing stem cells, which suggests that the self-adjustment of cells during the senescent process has enabled them to acquire a more aggressive growth potential, promoting tumor recurrence (155, 156). In CRC, activated heme oxygenase (HO-1) induced senescence escape of TIS cells after hemin treatment in IRINO exposed TIS cells, and escaped cells exhibited EMT and reproliferation similar to those after H_2_O_2_ treatment, which showed up-regulation of the antioxidant enzymes GPx-1 (157). These results suggest that the redox system, the state and complex regulation of ROS, and the acquisition of stemness in TIS cells are involved in the senescence escape of TIS tumor cells.

In another study, in TIS p53-null, p16-deficient human non-small cell carcinoma H1299 cells, a subset of cells could bypass replicative arrest and re-enter the cell cycle (158). Mechanistically, the aberrantly up-regulate Cdc2/Cdk1 promoted these cells escape from the senescence pathway. In another study, DOX effectively induced cellular senescence, resulting in cell cycle arrest at the G1 phase. This phenomenon was significantly correlated with cell colony formation following the release of DOX. Notably, cells pre-treated with DOX exhibited senescence-escaping cells. Furthermore, the combination of DOX with PD0332991 not only slowed down the progression of G1 phase cells but also enhanced cell colony formation (43). These findings suggest that the imbalance of cell cycle regulation after drug stimulation, which promotes the increase of cells in G1 phase with proliferative potential, is also one of the factors of senescence escape.

Interestingly, senescence escape was also observed in OIS cells. Escaped senescent cells can further facilitate tumor progression (159, 160). Approximately 80% of colorectal tumors harboring BRAF mutations (B-Raf-V600E) exhibit overexpression of the splice variant Rac1b. When B-Raf-V600E cells co-express with Rac1b, the senescent phenotype induced by BRAF was reversed. The expression of cell cycle inhibitors was down-regulated in an active oxygen species-dependent manner, sustaining the viability of CRC cells. These findings suggest that Rac1b overexpression provides a mechanism for CRC cells to escape BRAF-induced senescence and promote CRC progression (160, 161). Another research indicated that AP1 and POU2F2 transcription factors played a critical role in the senescent escape of CRC cells. POU2F2 expression is elevated in escaping cells in OIS, promoting senescence escape by increasing its binding affinity to cis-regulatory elements, and p16 is a critical target gene of POU2F2 (162).

## 7 Tumor dormancy and senescence

Tumor dormancy refers to a reversible long-term state in which tumor cells enter the G0/G1 phase, leading to an arrested capacity for proliferation (163). This process shares similarities with senescence, which is characterized by a permanent arrest of the cell cycle. Both states exhibit overlapping phenotypes and molecular pathways, including cell cycle arrest, reduced proliferation, resistance to apoptosis, immune evasion, elevated oxidative stress, metabolic dysregulation, and shared regulatory mechanisms involving p53, Rb, and cyclin-dependent kinase (CDK)-mediated cell cycle inhibition (164, 165). Distinct biomarkers can differentiate these two states. Dormant cells typically show a p38 MAPK^high^/ERK^low^ phenotype, elevated expression of the CDK inhibitor p27, and evidence of autophagy activation—an emerging feature of dormant cancer cells (165, 166). Dormant cells also retain the potential for reactivation, which can contribute to therapeutic resistance and the recurrence or metastasis of tumors (167–169). However, the concept of senescence escape has led some researchers to believe that senescence can be reversed, and senescent cells are a potential mechanism involved in cell dormancy (170, 171). After stress removal (for example, following treatment), residual cells may transition into either dormant or senescent states, highlighting the interconnected regulatory networks between them. Thus, dormant and senescent cells represent overlapping cellular states that share some markers but also have distinct differences. This raises the question: do tumors that reproliferate or emerge from recurrent lesions do so due to the escape of senescent cells or the awakening of dormant cells? To clarify this, more specific markers are necessary to distinguish between these states. Additionally, technical challenges remain in accurately detecting dormant cells, especially *in vivo*, and in developing models that differentiate dormancy from senescence after therapeutic interventions.

## 8 Conclusion and future direction

Cellular senescence is triggered by various stimuli, including spontaneous senescence or induced senescence. However, the dual role of cellular senescence in the pathogenesis and progression of CRC, as well as its potential impact on anti-tumor therapy, remains shrouded in great uncertainty. Elucidating the molecular mechanisms and regulatory networks underlying cellular senescence in CRC not only provides a theoretical foundation for understanding the key biological processes driving CRC initiation and progression, but also establishes a critical basis for developing innovative therapy strategies centered on cellular senescence.

The infection and distribution of intestinal microorganisms are associated with CRC and are important drivers of precancerous lesions in CRC (172). The senescence induced by bacterial infection has also become a new focus of attention. 2 Porphyromonas species (*P. asaccharolytica* and *P. gingivalis*) could induce cellular senescence by secreting bacterial metabolite butyrate, accelerating the occurrence of CRC (173). *Clostridium difficile* (*C. difficile*) produces genotoxins, generating highly genetically unstable cells, promoting the complete tumor transformation of tumor precancerous cells originating from the colon in CRC (174). These research perspectives offer a broader perspective for understanding the role of cellular senescence in CRC.

Apart from the senescence induced by traditional chemo- or radio-therapies, some traditional chinese medicine compounds and derivatives have also been noted for the role in inducing and eliminating cellular senescence, providing treatment opportunities for patients. For instance, the active substance Avenanthramide A (AVN A) extracted from oats can treat CRC by triggering cellular aging, and the miR-129-3p/Pirh2/p53 signaling pathway is involved in this process (175); the berberine derivative B68 has been proven to induce cellular senescence, disrupt the immunosuppressive PD-1/PD-L1 interaction, promote the rapid clearance of senescent tumor cells, and work synergistically with anti-CTLA4 therapy to further enhance anti-tumor immunity (176). β-Asarone (1-propenyl-2,4,5-methoxybenzol) is a compound from the traditional medical herb Acorus calamus Linn, it could inhibit the formation of CRC *in vivo* and *in vitro* by inducing senescence (177). Triterpenoids Cucurbitacin E (CE) induced CRC cellular senescence via modulating the miR-371b-5p/TFAP4 axis (178). Cyclovirobuxine (CVB-D) or artemisia annua derivative Artesunate inhibit the proliferation of CRC through induced senescence and autophagy (179, 180).

CRC exhibits heterogeneity, with distinct molecular subtypes demonstrating significant differences in clinical outcomes. Approximately 3% of mCRC patients exhibit deficient DNA mismatch repair (dMMR), resulting in high microsatellite instability (MSI-H) status that confers heightened sensitivity to immunotherapy, particularly immune checkpoint inhibitors (ICIs). In these MSI-H/dMMR mCRC patients, ICI treatment demonstrates significant tumor shrinkage and prolonged survival outcomes. However, the majority of mCRC cases are characterized by proficient mismatch repair (pMMR) and microsatellite stability (MSS), consequently exhibiting primary resistance to ICIs (181, 182). In clinical research, a phase II trial (NCT03800602 (144)) investigated the efficacy of nivolumab and metformin in patients with refractory MSS CRC, who were unresponsive to prior therapies. Although the combination treatment was well-tolerated in MSS CRC patients but showed no evidence of clinical efficacy. However, a trend toward T-cell infiltration was observed in tumors following dual treatment with metformin and PD-1 blockade. Therefore, senotherapy may be valuable in improving the treatment outcomes for patients with MSS. For refractory CRC, clinical trails have shown that metformin combined with chemotherapy drugs (fluorouracil, oxaliplatin or irinotecan) can demonstrate disease control (145, 146). However, more research evidence is still needed to provide the feasibility of senotherapy for improving the treatment outcomes of CRC.

There are still some issues that need to be paid attention to: 1) In the early stage of CRC development, cellular senescence plays a protective role by inhibiting further cell proliferation. However, the mechanism by which malignant transformed cells respond and overcome senescence during tumor development remains to be elucidated. 2) During CRC treatment, the concentration, metabolic rate, and half-life of chemotherapeutic agents critically determine their ability to induce cellular senescence. Moreover, the administration intervals for combination therapies incorporating senolytics or senomorphics require precise optimization. Thus, elucidating the precise mechanisms underlying drug-induced senescence is paramount for developing tailored treatment strategies. 3) The intricate and dynamic nature of the CRC microenvironment, where diverse factors interact to influence the induction and functionality of cellular senescence, necessitates the development of more sophisticated experimental models for comprehensive investigation. 4) In preclinical models, the phenomenon of senescence reversal or escape that has been observed is a matter that cannot be ignored, as it may be a significant factor contributing to poor clinical prognosis after cancer treatment. Therefore, a thorough understanding of the inducing factors and escape mechanisms of senescence is helpful in resisting this risk during treatment. 4) Lacking of specific senescence markers to detect and trace senescent cells *in vivo* is the challenge for utilizing senotherapy as a strategy for CRC treatment.

At present, there are no commercially available drugs on the market for treating cellular senescence. The most promising drug, navitoclax, is still in the third phase of clinical trials. The effects of metformin and rapamycin in combating cellular senescence in clinical trails are not yet fully confirmed. Therefore, our future direction is to determine the clinical anti - senescence effects of these drugs through clinical trials, and then bring them into clinical application, combined with current treatment methods, to improve the treatment effect of CRC. Overall, a deeper understanding of cellular senescence provides new insights and suggestions for the disease progression and treatment of CRC.These advancements hold significant scientific and clinical value for advancing precision medicine in CRC treatment.
